# Evaluation of Regional Geospatial Clusters in Inguinal Hernia Repair

**DOI:** 10.7759/cureus.26381

**Published:** 2022-06-27

**Authors:** Nicholas J Peterman, Richard L Li, Bradley D Kaptur, Eunhae G Yeo, Darrion Yang, Papus Keita, Kristine Carpenter

**Affiliations:** 1 Medicine, Carle Illinois College of Medicine, Urbana, USA; 2 Medicine, Carle Foundation Hospital, Urbana, USA; 3 Surgery, Carle Foundation Hospital, Urbana, USA; 4 Family Medicine, Carle Illinois College of Medicine, Urbana, USA; 5 Family Medicine, Carle Foundation Hospital, Urbana, USA

**Keywords:** geospatial analysis, open technique, laparoscopic technique, ambulatory surgical center, inguinal hernia repair

## Abstract

Introduction

There is significant variation in how inguinal hernia repairs are conducted across the United States (US). This study seeks to utilize national public data on inguinal hernia repair to determine regional differences in the use of ambulatory surgical centers (ASC) and in the choice of laparoscopic or open technique.

Methods

Medicare provider billing and enrollee demographic data were merged with US census and economic data to create a county-level database for the years 2014-2019. Location, technique, and total count of all inguinal hernia repair billing were recorded for 1286 counties. Moran’s I cluster analysis for inguinal hernia repairs, percent laparoscopic technique, and percent ACS were conducted. Subsequent hotspot and coldspot clusters identified in geospatial analysis were compared using ANOVA across 50 socioeconomic variables with a significance threshold of 0.001.

Results

There were 292,870 inguinal hernia repairs, of which 39.8% were conducted laparoscopically and 21.3% of which were in an ACS. Inguinal hernia repair coldspots were in the Mid-Atlantic and Northern Midwest, while hotspots were in Nebraska, Kansas, and Maryland (3.85 and 36.53 repairs per 1000 beneficiaries, respectively). Compared to coldspots, hotspot areas of repair were less obese, had less tobacco use, older, and less insured; there were no differences in gender, white population, or county urbanization (p<0.001). Laparoscopic technique coldspots were in the Mid-Atlantic, Michigan, and Great Plains, while hotspots were in the Rocky Mountains and contiguous states from Florida to Wisconsin (6.14% and 75.39%, respectively). ACS coldspots were diffusely scattered between Oklahoma and New Hampshire, while hotspots were in California, Colorado, Maryland, Tennessee, and Indiana (0.51% and 48.71%, respectively).

Conclusions

Inguinal hernia repair, the surgical setting, and the choice of technique demonstrated interesting geospatial trends in our population of interest that have not been previously characterized.

## Introduction

Inguinal hernia repair is a procedure done to repair outpouching of the abdominal viscera through weakened fascial planes. There are two forms of inguinal hernias: direct and indirect, which can present as unilateral, bilateral, or recurrent [1]. Direct hernias are often the result of increased abdominal pressure and gradual degeneration of the myofascial plain [1]. Indirect hernias involve herniation through the inguinal canal, which is often a consequence of a patent processus vaginalis or a sequelae of other urological conditions [1]. Inguinal hernia repairs are very common, with an estimated 800,000 repairs conducted annually [1]. Among adults, the risk of an inguinal hernia increases with age. As a result, Medicare databases are powerful tools for analyzing inguinal hernia repair, with literature as recent as 2019 identifying over 407,717 patients, amongst whom 87% were over the age of 65 [2]. Men are eight to 10 times more likely to develop an inguinal hernia, while race, obesity, connective tissue disorders, chronic cough, and constipation are additional risk factors for this disease [3]. Several of these risk factors-specifically obesity, age, and race-are well understood to have regional differences in prevalence [4-6]. If left untreated, patients are at risk for bowel necrosis due to strangulation, constituting a surgical emergency [7].

Treatment options for inguinal hernia repair include open hernia repair and laparoscopic hernia surgery. Currently, there is no consensus in the literature that suggests one method is superior to the other with respect to patient outcomes; however, it is suggested that laparoscopic repair has faster recovery, lower incidence of chronic pain, and is considered to be a cost-effective choice [8]. In comparing the two, a case-control study in patients over 60 years of age found that laparoscopic repair was superior with respect to intraoperative time, first peristalsis, defecation, analgesic duration, and hospital stay. In conducting the procedure, laparoscopic repair averaged 61 minutes per procedure, compared to 105 minutes for open repair [9]. However, prior studies suggest that open repair maintains lower incidences of urinary retention, overall complications, and inguinodynia [10]. For a surgeon to achieve a similar result as an open hernia repair using a laparoscopic technique, they must conduct between 50 and 100 procedures, with surgeon experience identified as a significant driver for good outcomes [11,12]. Finally, neither technique has a significant advantage in reducing recurrence rates [10,13]. 

Given the lack of sufficient data to suggest one method is superior to the other, the choice of repair type is nebulous. A survey on surgeon preference in hernia repair indicated that over 44% are influenced by their individual professional skills, another 44% base their decision on trends in the hospital, and 22% are based on patient preferences [14]. This could imply that for inguinal hernia repair, the decision between either option is driven primarily by an individual surgeon’s training and hospital decision-making. With the increasing prevalence of inguinal repairs conducted in the United States, there has been little to no quantification of where these procedures are being done and the communities most affected. The use of the Medicare provider database and the US Census allows for the capture of large quantities of patients undergoing inguinal hernia repair while simultaneously allowing for geospatial analysis to determine regional differences and characteristics. 

In the past two decades, hospital systems and independent providers have turned their attention toward the development of ambulatory surgery centers (ASCs). Between 1990 and 2011, ASCs have experienced explosive growth, nearly doubling with more than 5000 facilities. Total surgical center payments to ASCs experienced a 167% increase [15]. In 2015, the ASC market was valued at around $36 billion per year, with Medicare representing nearly $5 billion in facility payments to ASCs per year [16]. Hospital decision-making to construct ASCs can have a significant impact on the amount and type of hernia repairs performed in specific regions. There is very little literature on the national distribution of hernia repairs being conducted in the US, especially in the context of the rise of ASCs.

With physician training and hospital trends noted as the primary differentiators between repair methodology and several risk factors for hernias noted to have regional differences, the objective of this study is to determine regional differences in the use of laparoscopic or open hernia repair in the context of ambulatory surgical centers.

## Materials and methods

Multiple publicly available datasets were utilized in this paper, including Medicare Physician & Other Practitioners by Provider and Service dataset, Medicare Geographic Variation by National, State & County, and the Mapping Medicare Disparities by Population tool from the Center for Medicare Services (CMS) as well as socioeconomic data from the US Department of Agriculture (USDA) [17-20]. All datasets were obtained for the years 2014-2019 and, utilizing Python, were averaged across all years and merged on a county level. Counties were excluded from analysis if there was incomplete data across any of the aforementioned datasets. CMS physician billing data listed all Current Procedural Terminology (CPT) codes billed to Medicare during the time period of interest, as well as their location of service and provider type. Provider type was used to identify if a service was conducted at an ASC. CPT codes were used to filter the overall list to only the open inguinal hernia repairs (49505, 49507, 49520, 49521, and 49525) and the laparoscopic inguinal hernia repairs (49650 and 49651). The total number of inguinal hernia repairs was recorded for each county as well as what percent of them were conducted using laparoscopic technique. Total repairs were scaled per 1000 Medicare members in each county to account for differential population distributions. After further filtering of counties to remove those without billing of inguinal hernia repair during the time period, 1286 counties remained for final analysis, each with 37 recorded socioeconomic and inguinal hernia variables. 

Geospatial analysis using the Moran’s I statistic was subsequently conducted on the dataset using GeoDa, a statistical analysis program built for spatial clustering analysis. The Moran’s I value is calculated for each county for a single variable and compares each county’s value to that of the national average along with the counties’ neighbors’ values to the national average. Together, these comparisons allow Moran’s I analysis to identify statistically significant (p<0.05) clusters of a select variable and classify each county as either high-high, low-low, low-high, or high-low. The first “high” or “low” describes if a county is statistically significantly higher or lower than average. The second “high” or “low” describes if a county’s neighbors are statistically significantly higher or lower than the national average. If either a county or its neighbors are not statistically significantly different than the national average, then the county as a whole is not significant. High-high areas can be thought of as hotspots, while low-low areas are equivalent to coldspots. Low-high and high-low areas represent areas of significant special dissimilarity that often border hotspots and coldspots. Moran’s I was calculated for the percentage of procedures done in ASC, the total number of inguinal hernia surgeries per 1000 Medicare beneficiaries, and the percentage of procedures done laparoscopically. The resulting four statistically significant classifications were then used to group the counties, and ANOVA analysis was conducted across all 37 variables to determine if there exist statistically significant socioeconomic and surgical practice differences between clusters.

## Results

Two hundred ninety-two thousand eight hundred seventy inguinal hernia repairs between 2014 and 2019 were included in this study. Of these procedures, 21.3% were performed in an ASC, and 39.8% were conducted laparoscopically. 

**Figure 1:**
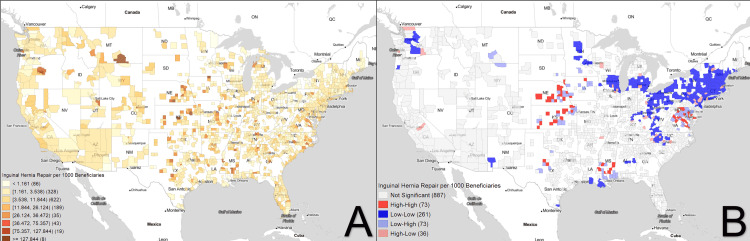
Inguinal hernia repair per 1000 beneficiaries Figure 1A shows the choropleth map distribution of inguinal hernia repair while Figure 1B displays the corresponding Moran's I plot. White areas correspond to counties excluded from analysis due to lack of any repairs in the time frame of interest.

**Figure 2:**
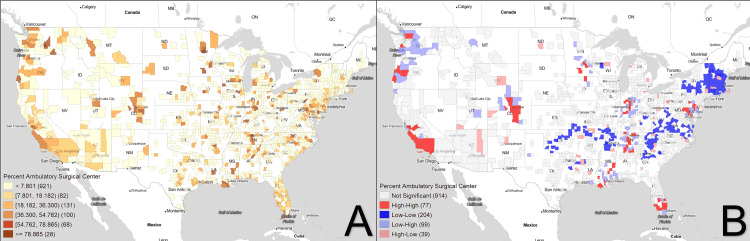
Percent ambulatory surgical center Figure 2A shows the choropleth map distribution of the percent of inguinal hernia repairs conducted in an ambulatory surgical center per county while Figure 2B displays the corresponding Moran's I plot. White areas correspond to counties excluded from analysis due to lack of any repairs in the time frame of interest.

**Figure 3:**
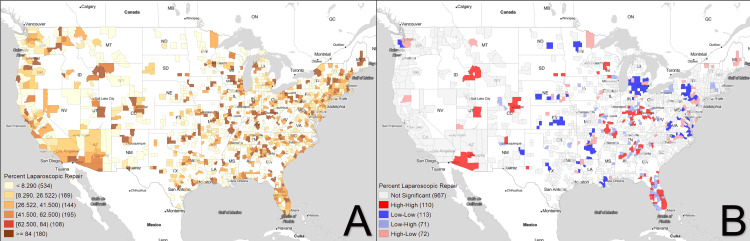
Percent laparoscopic repair Figure 3A shows the choropleth map distribution of the percent of inguinal hernia repairs conducted using laparoscopic technique while Figure 3B displays the corresponding Moran's I plot. White areas correspond to counties excluded from analysis due to lack of any repairs in the time frame of interest.

Figures 1-3 demonstrate the geospatial distribution of inguinal hernia repair, percent ambulatory surgical centers, and percent laparoscopic repairs. With respect to distribution alone, areas of high prevalence for inguinal hernia repair include regions in Montana, Oregon, California, and several scattered hotspots in the Midwest. ASCs seem to have a greater focus on population centers, with clusters identified in more populous regions per state. For example, in Illinois, ASCs have a much larger density in regions surrounding Chicago than in less populated areas. Percent laparoscopic repair seems to follow a similar trend, with areas with larger population densities having greater amounts of laparoscopic repair than more rural counties. 

Overall, 261 counties were identified as coldspots for inguinal hernia repair and were concentrated in the Eastern US and the Pacific Northwest. Seventy-three counties were identified as hotspots and were localized to the Midwest region, specifically Nebraska and Kansas, with an additional hotspot in the South, in Mississippi. The rise of ambulatory surgical centers seems to be much more specific, with 77 counties identified as hotspots in California, Oregon, Washington, and Florida. Two hundred four coldspots were identified, seemingly concentrated in the Northeastern US. Percent laparoscopic repair has notable hotspots in Arizona, Florida, Idaho, Utah, Florida, and the Midwest, with specific coldspots appearing in Michigan and the Eastern US. 

Table 1 demonstrates the results of an ANOVA performed across the respective permutations of high and low groupings in Moran I analysis for inguinal hernia repair per 1000 beneficiaries. Age, African American race, education, alcohol abuse, asthma, chronic obstructive pulmonary disease (COPD), depression, drug abuse, and tobacco use were significantly different between groups. Comparing hotspots to coldspots, hotspots had, on average, higher Medicare ages (72.64 to 70.89), percent Black race (9.96% to 6.97%), and college attainment (28.62% to 32.31%). Yet interestingly, they had lower rates of alcohol abuse (1.39% to 2.39%), asthma (4.47% to 5.36%), COPD (10.91% to 12.53%), tobacco use (7.82% to 10.38%), and depression (16.69% to 19.58%). 

**Table 1:** ANOVA analysis of inguinal hernia repair per 1000 beneficiaries COPD - chronic obstructive pulmonary disease

ANOVA analysis of inguinal hernia repair per 1000 beneficiaries									
Cluster	High-high		Low-low		Low-high		High-low		p-value
Counties per cluster	73		261		73		36		
Demographic variable	Mean	SD	Mean	SD	Mean	SD	Mean	SD	
Average Medicare age	72.46	1.4	70.89	1.5	71.93	1.5	71.38	1.89	7.88x10^-15^
% Male	44.68	1.51	45.42	1.76	44.81	1.84	45.67	2.26	1.16x10^-3^
% White	83.84	11.22	88.16	10.41	83.89	13.24	87.87	8.13	2.06x10^-3^
% Black	9.96	11.39	5.6	6.97	10.19	12.78	4.65	4.32	9.62x10^-6^
% Hispanic	2.55	2.91	2.76	4.67	2.58	3.93	3.38	5.12	7.96x10^-1^
% Other Race	3.64	2.82	3.47	2.07	3.34	2.62	4.1	2.43	3.97x10^-1^
Medicare population density	101.07	201.19	185.83	795.82	63.88	149.18	133.29	297.4	4.42x10^-1^
Total population density	883.62	2014.47	1244.54	5639.2	424.06	993.05	909.85	2376.28	5.66x10^-1^
Metro (binary value 0,1)	0.56	0.5	0.68	0.47	0.67	0.47	0.69	0.47	2.90x10^-1^
Urban (binary value 0,1)	0.21	0.41	0.28	0.45	0.18	0.39	0.25	0.44	2.95x10^-1^
% Poverty	12.72	6.04	13.43	4.61	12.27	5.11	13	7.31	3.51x10^-1^
Median household income in US dollars	60910.6	19575.92	57723.52	13470.12	61784.64	18159.36	64634.46	23297.6	3.64x10^-2^
Unemployment	3.96	1.78	4.87	1.18	4.11	1.23	4.92	1.55	3.17x10^-8^
Uninsured	11.13	3.81	7.91	2.92	10.37	3.77	8.65	3.81	2.79x10^-14^
Rural Urban Continuum Code (integer 1-9, 1=most urban, metro<=3)	4.04	2.72	2.92	1.98	3.27	2.22	3.22	2.55	1.99x10^-3^
% Without a high school diploma	10.75	6.1	10.65	4.69	10.4	4.59	11.38	6.74	8.2564x10^-1^
% With only a high school diploma	28.62	7.77	32.31	7.47	29.37	6.21	29.12	7.88	9.01x10^-5^
% Some college	30.51	6.79	28.59	4.25	31.1	4.43	27.71	5.8	4.36x10^-5^
% College degree	30.11	14.28	28.46	10.65	29.14	10.99	31.81	13.86	3.50x10^-1^
% Medicare alcohol abuse	1.57	0.55	2.39	0.68	1.79	0.65	2.08	0.68	3.00x10^-22^
% Medicare asthma	4.47	1.15	5.36	1.16	4.49	0.9	4.96	0.9	3.49x10^-12^
% Medicare chronic kidney disease	20.13	2.78	21.56	3.49	20.67	2.79	21.22	3.33	5.51x10^-3^
% Medicare COPD	10.91	2.31	12.53	3.22	11.23	2.34	11.99	3.85	6.69x10^-5^
% Medicare depression	16.69	2.48	19.58	2.95	17.69	2.91	18.17	2.94	4.06x10^-14^
% Medicare diabetes	26.02	3.86	26.91	4.54	26.44	3.64	26.27	3.74	3.83x10^-1^
% Medicare drug abuse	2.07	1.59	3.05	1.08	2.42	1.35	2.76	1.5	1.31x10^-8^
% Medicare osteoporosis	6.3	1.66	6.1	1.32	5.95	1.28	6.39	1.35	2.93x10^-1^
% Medicare stroke	3.42	0.76	3.63	0.66	3.56	0.78	3.54	0.61	1.56x10^-1^
% Medicare tobacco use	7.82	2.1	10.38	3.09	8.55	2.38	9.58	4.32	6.14x10^-11^
% Medicare chronic pain/fibromyalgia	17.67	2.67	17.67	2.86	18.16	2.59	18.31	2.7	3.66x10^-1^
% Medicare migraine	2.52	0.64	3	0.6	2.78	0.6	2.91	0.57	6.57x10^-8^
% Medicare obesity	13.82	3.91	17.71	5.04	14.81	3.74	15.73	4.31	6.94x10^-11^
Inguinal hernia repair per 1000 beneficiaries	36.53	34.16	3.85	2.6	5.33	2.7	18.05	9.87	1.22x10^-51^
Open repair per 1000 beneficiaries	27.39	27.82	2.4	2.11	3.85	2.83	11.31	8.95	1.63x10^-45^
Laparoscopic repair per 1000 beneficiaries	9.14	20.12	1.45	1.93	1.47	2.02	6.73	6.46	2.39x10^-11^
% Ambulatory surgical center	16.99	25.89	10.55	22.49	9.08	21.42	12.76	20.1	1.31x10^-1^
% Hospital	83.01	25.89	89.45	22.49	90.92	21.42	87.24	20.1	1.31x10^-1^
% Open repair	76.08	26.98	66.21	34.52	73.05	36.06	62.67	31.09	6.11x10^-2^
% Laparoscopic repair	23.92	26.98	33.79	34.52	26.95	36.06	37.33	31.09	6.11x10^-2^

For procedures conducted at ASC, comparing hotspots to coldspots using ANOVA, median household income, general educational diploma (GED) status, osteoporosis, tobacco use, repairs per 1000 beneficiaries, type of repair, and location were significantly different among others represented in Table 2. Of note, hotspots for repairs conducted at ASC have higher median household incomes ($62,294 to $51,855), lower rates of individuals with only a GED (27.93% to 32.32%), and conducted both more open and laparoscopic repairs (11.29 to 5.83 and 5.22 to 2.67 per 1000 beneficiaries, respectively). Hotspots had over 48.3% of procedures conducted at ambulatory surgical centers, while in coldspots, only 0.52% were conducted at an ASC. Of note, both hotspots and coldspots have similar rates of open and laparoscopic repair rates (66.80 to 66.82, and 33.20 to 33.26 per 1000 beneficiaries, respectively).

**Table 2:** ANOVA analysis of ambulatory surgical center clusters COPD - chronic obstructive pulmonary disease

ANOVA analysis of ambulatory surgical center clusters									
Cluster	High-high		Low-low		Low-high		High-low		p-value
Counties per cluster	77		204		99		39		
Demographic variable	Mean	SD	Mean	SD	Mean	SD	Mean	SD	
Average medicare age	71.49	1.51	71.01	1.36	71.05	1.53	70.88	1.1	5.60x10^-2^
% Male	45.36	1.73	45.24	1.84	45.96	1.99	45.43	1.52	1.62x10^-2^
% White	83.21	10.98	86.13	11.67	85.72	12.82	84.34	11.79	2.97x10^-1^
% Black	7.71	7.93	9.46	11.7	6.84	10.67	9.92	11.49	1.77x10^-1^
% Hispanic	4.77	6.78	1.4	1.65	4.22	7.78	2.41	4.3	3.54x10^-7^
% Other Race	4.31	4.07	3.02	2.26	3.21	2.44	3.33	5.42	2.24x10^-2^
Medicare population density	78.83	95.37	75.74	158.61	47.18	121.1	48.78	68.34	2.24x10^-1^
Total population density	523.49	733.73	457.09	1195.27	293.04	825.45	252.41	364.11	2.75x10^-1^
Metro (binary value 0,1)	0.78	0.42	0.53	0.5	0.59	0.49	0.69	0.47	1.09x10^-3^
Urban (binary value 0,1)	0.29	0.46	0.17	0.38	0.09	0.29	0.13	0.34	5.02x10^-3^
% Poverty	13.1	4.88	15.43	5.18	13.91	5.15	15.94	5.05	1.21x10^-3^
Median household income in US dollars	62294.12	18216.08	51855.72	13236.94	56875.98	15889.8	50558.83	10703.91	6.42x10^-7^
Unemployment	4.46	1.23	4.77	1.2	4.87	1.41	4.97	1.27	1.06x10^-1^
Uninsured	10.29	4.16	11.1	4.39	10.37	3.69	11.42	4.09	2.54x10^-1^
Rural Urban Continuum Code (integer 1-9, 1=most urban, metro<=3)	2.72	2	3.81	2.37	3.58	2.32	3.41	1.79	4.80x10^-3^
% Without a high school diploma	10.86	5.22	12.3	4.8	12.35	5.66	11.97	4.47	1.74x10^-1^
% With only a high school diploma	27.83	8.79	32.32	7.02	31.5	8.53	31.02	5.64	3.01x10^-4^
% Some college	29.33	5	28.89	4.09	30.67	4.83	31.02	4.01	2.03x10^-3^
% College degree	31.98	12.85	26.49	11.36	25.48	12.1	25.99	8.44	1.15x10^-3^
% Medicare alcohol abuse	2.12	0.72	2.2	0.72	1.98	0.64	2.17	0.63	8.98x10^-2^
% Medicare asthma	4.81	0.77	4.94	1.17	4.71	1.19	4.69	1.04	2.78x10^-1^
% Medicare chronic kidney disease	21.49	3.53	21.26	3.25	20.7	3.99	20.72	3.29	3.77x10^-1^
% Medicare COPD	11.03	3.18	12.78	3.22	11.94	3.82	12.18	2.73	1.32x10^-3^
% Medicare depression	18.14	2.79	18.85	2.81	18.37	3.34	18.65	2.68	2.78x10^-1^
% Medicare Diabetes	25.64	5.2	26.98	4.08	26.13	5.2	26.27	4.25	1.38x10^-1^
% Medicare drug abuse	3.45	1.54	2.99	1.02	3.46	1.45	2.95	0.88	2.12x10^-3^
% Medicare osteoporosis	6.3	1.39	5.66	1.23	5.62	1.41	6.21	1.17	2.97x10^-4^
% Medicare stroke	3.55	0.76	3.51	0.62	3.54	0.94	3.61	0.81	8.65x10^-1^
% Medicare tobacco use	8.41	2.75	10.45	2.77	9.54	3.15	10.09	2.58	2.61x10^-6^
% Medicare chronic pain/fibromyalgia	18.6	2.45	17.86	2.79	18.92	3	18.53	2.5	1.12x10^-2^
% Medicare migraine	3.06	0.55	2.9	0.55	2.87	0.54	2.93	0.51	9.69x10^-2^
% Medicare obesity	15.01	3.96	15.67	3.59	15.31	4.72	16.63	5.11	2.10x10^-1^
Inguinal hernia repair per 1000 beneficiaries	16.51	29.69	8.5	10.92	6.14	6.1	17.05	17.31	6.47x10^-6^
Open Repair per 1000 Beneficiaries	11.29	26.24	5.83	9.51	4.12	5.92	11.48	12.13	7.87x10^-4^
Laparoscopic repair per 1000 beneficiaries	5.22	9.74	2.67	4.57	2.02	3.14	5.57	10.67	5.47x10^-4^
% Ambulatory surgical center	48.3	21.6	0.52	1.97	0.47	2	36.22	20.81	1.45x10^-124^
% Hospital	51.7	21.6	99.48	1.97	99.53	2	63.78	20.81	1.45x10^-124^
% Open repair	66.8	30.26	66.82	35.98	64.69	40.36	72.77	24.94	6.89x10^-1^
% Laparoscopic repair	33.2	30.26	33.18	35.98	35.31	40.36	27.23	24.94	6.89x10^-1^

## Discussion

Previous studies have suggested that surgeon preference in decision-making between open and laparoscopic inguinal repair is driven by surgeon training [21] and hospital trends [14]. This study has identified regional differences in which one method is preferred over another using Medicare and US Census data. 

As demonstrated in Figure 1, the hotspots for inguinal hernia repair included the states of Nebraska, Kansas, and Mississippi. These states endorse some of the highest rates of obesity in the US at 32%, 31.2%, and 37.3%, respectively [22]. Mississippi further experiences high incidences of alcoholism and tobacco use compared to the rest of the country. On cluster analysis, there seems to be no specific relationship between population size and inguinal repair. Specifically, the Northeastern United States is a notable coldspot for inguinal repair while being among the most populous portion of the country. A possible explanation may be the relative good health of adults, with states like New York, New Jersey, and Rhode Island having some of the lowest obesity rates and tobacco use while endorsing some of the highest per capita public health funding and exercise rates in the country [23]. In addition, prior studies have suggested a link between occupation and the prevalence of inguinal repairs. A study by Vad et al. found that occupational mechanical exposures in men between the ages of 15-65 increase the risk of lateral inguinal hernia repair and could be prevented in approximately 15% of cases [24]. This aspect may further explain increased incidences of hernia repair in states known for manufacturing and industry. For instance, Mississippi, a hotspot for hernia repair, reports the highest concentrations of upholsterers, fallers, and logging equipment operators (all of which are high-risk, labor-intensive industries), while New York supports the highest concentration of fashion designers, advertising and promotion managers, and fabric and apparel patternmakers [25]. 

This geospatial analysis did not find urbanization and poverty to be significant in comparison between coldspots and hotspots. This is supported by one prior single-institution study that found no relationship between a low socioeconomic status and presentation of an inguinal hernia [26]. In relation to the established risk factors for inguinal hernias, this study finds that age, tobacco, alcohol, and conditions with chronic cough are paradoxically correlated with regions that have reduced inguinal hernia surgeries. Overall, in comparing hotspots to coldspots, it appears that hotspots tend to have lower incidences of drug abuse, tobacco use, and COPD yet perform the most inguinal hernia repairs, as seen in Table 1. This may be due to the availability of care being concentrated in a select few centers, with access to care being an identified issue in coldspots. The uneven distribution of healthcare has been a well-studied phenomenon, with a recent 2022 study finding that patients in rural settings have worse health status yet lower healthcare utilization for both primary and specialty care [27]. To make matters worse, since January 2005, 181 rural hospitals have closed, with the rate of closures only increasing [28,29]. 

This study further demonstrates the regional differences in the surgical setting in which these repairs are conducted. With surgery often divided into two competing markets, outpatient and inpatient, the majority of surgical procedures done in the US are currently conducted in outpatient settings. Many large hospital groups are investing in the development of ASCs as part of their future business plans [30]. This study finds ASCs to be concentrated in California, the Pacific Northwest, and the Midwest. The initial expansion of ASCs was driven by high demand, with organizations pushing for larger surgical centers [16]. However, in 2008, ASC supply immediately ceased to increase, and the number of ASCs entering the market began to dramatically decrease. This may have been due to the abrupt transition in the Medicare fee schedule transition that led to slimmer profit margins and reduced investment in new construction [31]. With lower profits and the requirement for higher volume, new ASC construction in expensive and population-dense regions of the US quickly became a more risky business plan. Another option is to construct ASCs in regions where patients may be willing to pay for more outpatient procedures. California, a notable hotspot in this study, with a total of 817 ASCs, has ophthalmology, orthopedics, and pain specialists representing over 42% of all single-specialty ASCs [32]. The profitability of Californian ASCs may be the reason for their prevalence, maintaining an operating margin between 26-28.6% between the years 2012 and 2016, and up to 40.8% of patients using private insurance [33]. However, the exact reason for why certain counties across multiple states support a high density of ASCs is unknown and is most likely multifactorial. 

With regard to laparoscopic technique, coldspots were localized in the Mid-Atlantic, Michigan, and Great Plains, while hotspots were predominantly in a series of states stretching from Florida to Wisconsin and the Rocky mountains. Laparoscopic technique correlates remarkably well with the 2021 US Bureau of Labor Statistics location quotient for general surgeons [34]. Areas with high availability of general surgeons have higher levels of laparoscopic procedures. This suggests that laparoscopic procedures are currently limited to specific metropolitan areas, and rural communities may not be able to enjoy the same level of access. 

Limitations

The strength of this study is in the unprecedented number of inguinal surgeries captured in the analysis and the novel geospatial approach in which national trends are identified with a spatial granularity not seen in the existing literature. With regard to the limitations of this study, it must be acknowledged that this study excluded areas with no inguinal hernia repairs performed. As a result, rural counties were preferentially removed from the analysis. This may introduce bias that favors the analysis of areas with relative access compared to those with no access. Further, Medicare data, while an effective representation of some aspects of the US health system, is only limited to Medicare beneficiaries and thus does not capture the entire population of healthcare users. 

## Conclusions

This study characterizes the regional differences between the choice of technique for inguinal repair, local population differences, and the prevalence of ASC. Unique to this investigation is the level of granularity achieved in utilizing Medicare provider billing data merged with US census and economic data to provide county-level information on this particular procedure. This study finds inguinal hernia repair to have the greatest incidence in Nebraska, Kansas, and Maryland, with laparoscopic technique more often performed in regions with higher concentrations of general surgeons. As ASCs continue to expand, this study finds that the majority of ASCs are localized in regions such as California, Colorado, Maryland, Tennessee, and Indiana. Profitability may be the predominant driving factor for construction, with the patient population and insurance type as key elements. 

Further studies will include identifying trends over time and the effect of ongoing changes in Medicare reimbursement in inguinal hernia repairs and the locations for which they’re performed. In addition, transitioning to the usage of zip code rather than the county would further increase data granularity and may allow for better identification of regions with poor access to care to inform healthcare outreach with such initiatives as increased Medicare reimbursements or transportation discounts.
